# Mrgprb4-Lineage Neurons Participate in the Intervention of TENS Effects on Chronic Pain and Anxiety-like Symptoms in an Inflammatory Pain Mouse Model

**DOI:** 10.3390/biomedicines14030670

**Published:** 2026-03-15

**Authors:** Longhua Du, Hongyi Cheng, Jiamian Zhang, Hang Sun, Xia Li, Shuya Wang, Yun Liu, Bing Zhu, Xinyan Gao, Kun Liu

**Affiliations:** 1 Institute of Acupuncture & Moxibustion, China Academy of Chinese Medical Sciences, Beijing 100700, China; 18232752632@163.com (L.D.); 18779367324@163.com (H.C.); 13729251323@163.com (J.Z.); yangu13001594670@163.com (H.S.); xiali@bucm.edu.cn (X.L.); wagnshuya@mail.cintcm.ac.cn (S.W.); rowone268@126.com (Y.L.); zhubing@mail.cintcm.ac.cn (B.Z.); 2 Institute of Integrated Traditional Chinese and Western Medicine, West China Hospital of Sichuan University, Chengdu 610041, China

**Keywords:** transcutaneous electrical nerve stimulation, Mrgprb4-lineage neurons, chronic pain, anxiety, Complete Freund’s Adjuvant

## Abstract

**Background**: Mas-related G-protein-coupled receptor b4 (Mrgprb4)-lineage neurons in the peripheral nervous system are a type of C fibers in hairy skin. Our prior work demonstrated that these neurons respond to both noxious and innocuous mechanical and thermal stimuli. Ablating them eliminates the pleasant sensation elicited by gentle pressure on a mouse’s nape. However, their potential role in mitigating pain and pain-related negative emotions in response to somatic stimuli remains unclear. **Methods**: A CFA-induced chronic pain and anxiety comorbidity model was established in C57BL/6J mice. In vivo calcium imaging of dorsal root ganglia (DRG) neurons in *Mrgprb4*-GCaMP6s transgenic mice characterized neuronal responses to transcutaneous electrical nerve stimulation (TENS) at the Zusanli (ST36) acupoint. Optogenetic activation (Mrgprb4-ChR2 mice) and viral ablation of Mrgprb4-lineage neurons were employed to evaluate their role in mediating TENS effects on mechanical pain thresholds and anxiety-like behaviors. **Results**: In vivo calcium imaging revealed that 0.5 mA TENS preferentially activated Mrgprb4-lineage neurons compared to 2.0 mA TENS. In CFA model mice, 0.5 mA TENS at ST36 significantly increased mechanical pain thresholds and reduced anxiety-like behaviors in the open-field test. Optogenetic activation of Mrgprb4-lineage neurons at ST36 replicated these analgesic and anxiolytic effects, demonstrating the sufficiency of these neurons for therapeutic outcomes. Conversely, viral ablation of L3–L5 Mrgprb4-lineage neurons substantially attenuated the therapeutic effects of 0.5 mA TENS for both pain relief and anxiety reduction, indicating their necessity in mediating TENS efficacy. **Conclusions**: Mrgprb4-lineage neurons serve as critical peripheral mediators of TENS-induced analgesia and anxiolysis. These findings identify a specific neuronal population underlying the therapeutic effects of somatic stimulation at ST36, providing mechanistic insights that may guide optimization of TENS parameters for treating chronic pain and comorbid anxiety in clinical settings.

## 1. Introduction

Pain represents a grave global public health challenge, severely threatening human well-being. Chronic pain afflicts 37% of the population in developed nations and 41% in developing ones, with over half of sufferers experiencing emotional comorbidities, particularly anxiety, creating a vicious cycle that severely undermines patients’ physical and mental quality of life [1,2,3,4,5]. A deeper understanding of the neural mechanisms driving this comorbidity is crucial to uncovering novel therapeutic directions for chronic pain. Previous research has demonstrated that the majority of C-afferents are polymodal nociceptors actively involved in pain transmission. Furthermore, ablation of mas-related G-protein-coupled receptor D neurons reduces behavioral sensitivity to noxious mechanical stimuli in mice [6,7,8]. After knocking out the transient receptor potential vanilloid 1 (TRPV1) channel, mechanical hyperalgesia in arthritis model mice is alleviated [9]. Meanwhile, electroacupuncture (EA) alleviates pain in Complete Freund’s Adjuvant (CFA) model mice specifically through the TRPV1 signaling pathway in the brain [10,11].

Our previous research has revealed that mas-related G-protein-coupled receptor b4 (Mrgprb4)-lineage neurons represent a functionally distinct subpopulation of unmyelinated C-fibers that specifically innervate hairy skin [12,13]. These neurons are characterized by their polymodal sensory properties, responding to both innocuous and noxious mechanical stimuli as well as thermal stimulation [12]. Critically, Mrgprb4-lineage neurons are indispensable for transmitting pleasant sensations evoked by gentle stroking and caressing touch, distinguishing them from classical nociceptors [12,13]. In vivo calcium imaging studies have demonstrated that these neurons encode the velocity and force of pleasant tactile stimuli, suggesting their role as specialized mechanoreceptors for affective touch [12]. Furthermore, Mrgprb4-lineage neurons are functionally connected to the brain’s reward circuitry; they are required for dopamine release in the nucleus accumbens during social and sexual behavior in female mice, indicating their involvement in positive emotional states [14]. Notably, genetic ablation of Mrgprb4-lineage neurons does not alter baseline pain thresholds, motor function, or provoke spontaneous pain behaviors, underscoring their selective role in pleasant sensation rather than nociception [14]. These findings position Mrgprb4-lineage neurons as unique candidates for mediating the therapeutic effects of somatic stimulation.

In current clinical practice, a combination therapy of analgesics and anti-anxiety/depression drugs is widely adopted for managing chronic pain comorbid with anxiety [15]. While this regimen can mitigate patients’ pain and associated negative emotions to some degree, its efficacy remains dose-dependent and is accompanied by notable adverse reactions, including gastrointestinal and hepatorenal function impairments, as well as drug dependence [16]. Consequently, there is an urgent need to develop safer, more effective therapeutic strategies for the comorbidity of chronic pain and anxiety and to further elucidate the underlying therapeutic targets. The analgesic effect of transcutaneous electrical nerve stimulation (TENS) is well established, demonstrating significant efficacy in relieving diverse chronic pain conditions such as inflammatory pain, low back pain, postoperative incisional pain, and fibromyalgia [17,18,19,20,21,22]. It produces acupuncture-like effects with distinct advantages and minimal obvious adverse reactions. Furthermore, as an emerging physical therapy modality, TENS has demonstrated unique potential for intervening in anxiety and depression [23]. Previously published studies have established its efficacy in treating anxiety disorders during and after surgery [24,25,26]; however, evidence supporting TENS’s anti-anxiety effects from basic research remains limited [26]. Zusanli (ST36) is a prominently selected point for managing pain syndromes and emotional disorders. Two research studies have demonstrated that TENS or EA applied at ST36 significantly reduces postoperative opioid analgesic requirements and elevates TRPV1 expression levels to attenuate pain and depression [11,27]. Mrgprb4-lineage neurons transmit pleasant sensations induced by mild pressure and could represent key peripheral polymodal receptors, enabling TENS function [12]. Whether the mechanism underlying TENS alleviation of anxiety-like behaviors accompanying chronic pain involves Mrgprb4-lineage neurons remains unknown.

Despite accumulating evidence that Mrgprb4-lineage neurons mediate pleasant touch and interface with reward circuitry, their potential involvement in TENS-induced analgesia and anxiolysis remains unexplored. Given that TENS at ST36 produces therapeutic effects through peripheral sensory activation, that Mrgprb4-lineage neurons respond to gentle mechanical stimulation similar to TENS parameters, that these neurons project to brain regions implicated in both pain modulation and emotional regulation, and that their activation is associated with positive affective states, we hypothesized that Mrgprb4-lineage neurons serve as critical peripheral mediators of TENS therapeutic effects in chronic pain and anxiety comorbidity. To test this hypothesis, complementary experimental approaches were employed, combining in vivo calcium imaging, optogenetic manipulation, and viral ablation strategies in genetically engineered mouse models. By establishing causal relationships between Mrgprb4-lineage neuron activity and TENS efficacy, this study aims to identify specific neuronal populations underlying acupoint-based therapies, elucidate peripheral mechanisms of pain–anxiety comorbidity modulation, and provide mechanistic insights to optimize TENS parameters for clinical translation.

## 2. Materials and Methods

### 2.1. Animals

All animal experiments strictly adhered to the National Institutes of Health Guide for the Care and Use of Laboratory Animals. The Animal Ethics Committee at the Institute of Acupuncture and Moxibustion, China Academy of Chinese Medical Sciences, rigorously reviewed and formally approved all experimental protocols.

We conducted experiments using C57BL/6J mice (obtained from SPF Biotechnology Co. Ltd., Beijing, China, License No: SCXK-[Jing]-2019-0010). Mrgprb4^Cre-tdTomato^ transgenic strains were generated by Shanghai Model Organisms Center, Inc. (Shanghai, China). Mouse lines of Ai96 (RCL-GCaMP6s mice) (no. 028866) and Ai32 (RCL-ChR2/EYFP mice) (no. 024109) were purchased from The Jackson Laboratory (Bar Harbor, ME, USA). Sample sizes were determined using G*Power 3.1 (Heinrich Heine University, Düsseldorf; F tests, ANOVA: repeated measures, within–between interaction; effect size f = 0.40, α = 0.05, power = 0.80, 4 groups, 4 time points, correlation among repeated measures = 0.5, nonsphericity correction ε = 1). An a priori analysis indicated a minimum total sample size of 16 (4 per group). Accordingly, *n* = 12 mice per group was used for all behavioral experiments; post hoc power analysis confirmed an achieved power of 0.9999 (total *N* = 48). For viral ablation experiments, *n* = 10–12 per group was used; post hoc analysis confirmed an achieved power of 0.9998 (total *N* = 40, conservative estimate). For in vivo Ca^2+^ imagin, *n* = 5 mice was used, consistent with established DRG calcium imaging protocols [28]. Inclusion criteria were (1) age 8 weeks at experiment start, both male and female; (2) body weight 22–25 g; and (3) baseline mechanical threshold within the normal range (von Frey). Exclusion criteria: animals that failed to develop signs of inflammatory pain (paw swelling and mechanical hypersensitivity) following CFA injection.

Housed within standard animal facilities, mice were given ad libitum access to food and water. They experienced a consistent 12 h light–dark cycle (dark phase: 8:00 pm to 8:00 am), with ambient temperature maintained at 23 °C ± 0.5 °C, humidity controlled between 60% and 70%, and environmental noise kept below 60 dB. All animals acclimated to these conditions for seven full days prior to experimentation. Crucially, all experiments were performed by experimenters rigorously blinded to the specific genotype of the mice.

A mouse model of chronic pain and anxiety comorbidity (designated CFA mice) was successfully established via subcutaneous administration of Complete Freund’s Adjuvant (CFA) into the hind paw. Under isoflurane inhalation anesthesia, precisely 25 μL of CFA was meticulously injected subcutaneously into the right hind paw of each mouse. The needle penetrated approximately 0.5 cm deep, and the injection process deliberately spanned over 30 s. Upon needle withdrawal, the injection site was promptly compressed with a sterile cotton ball to prevent solution leakage. Following CFA injection, the hind paw exhibited marked redness and swelling, accompanied by distinct behavioral manifestations like persistent foot shaking, vigorous licking, and pronounced lameness; concurrently, the mechanical pain threshold showed a significant reduction. Control mice received an equal volume (25 μL) of sterile 0.9% saline injected into the right hind paw using an identical protocol. After thorough disinfection with iodophor, all mice were carefully returned to their home cages for attentive post-procedural care.

### 2.2. Intrathecal Injection

Mrgprb4-Cre; GCaMP6s mice were anesthetized by intraperitoneal administration of 1.25% tribromoethanol (0.2 mL/10 g body weight; Aibei Biotechnology, Nanjing, China). Core body temperature was maintained at approximately 37 °C using a heating pad. A 1 cm skin incision was made over the lumbar region to expose the T11–T12 intervertebral space. Under stereomicroscopic guidance (OLYMPUS SZ51, Tokyo, Japan), the intervertebral membrane and underlying dura mater were carefully exposed using fine forceps. A 0.2 mm diameter catheter connected to a microinjection system was stereotaxically inserted 5 mm caudally into the T11–T12 intervertebral space. Subsequently, 5 μL of either AAV9 (rAAV-CMV-DIO-taCasp3-T2A-TEVp; BrainCase Inc, Shenzhen, China., BC-0128, 1.1 × 10^13^ GC/mL) or sterile 0.9% saline was infused into the intrathecal space at a constant rate of 1.2 μL/min. The surgical incision was closed 10 min post-injection. Animals were monitored during postoperative recovery. Behavioral assessments were conducted ≥3 weeks following intrathecal injection.

### 2.3. Measurement of Hind Paw Thickness

Hind paw thickness measurements were obtained using a digital caliper. To prevent stress responses from compromising experimental outcomes, paw thickness was assessed while mice were carefully maintained under 0.5–1% isoflurane anesthesia (R510-22-10, RWD). The mouse’s hind paw was gently positioned horizontally. The operator then precisely aligned the caliper jaws with the peak swelling point on the dorsum of the foot and recorded the measurement.

### 2.4. Mechanical Pain Threshold Detection

**Figure 1:**
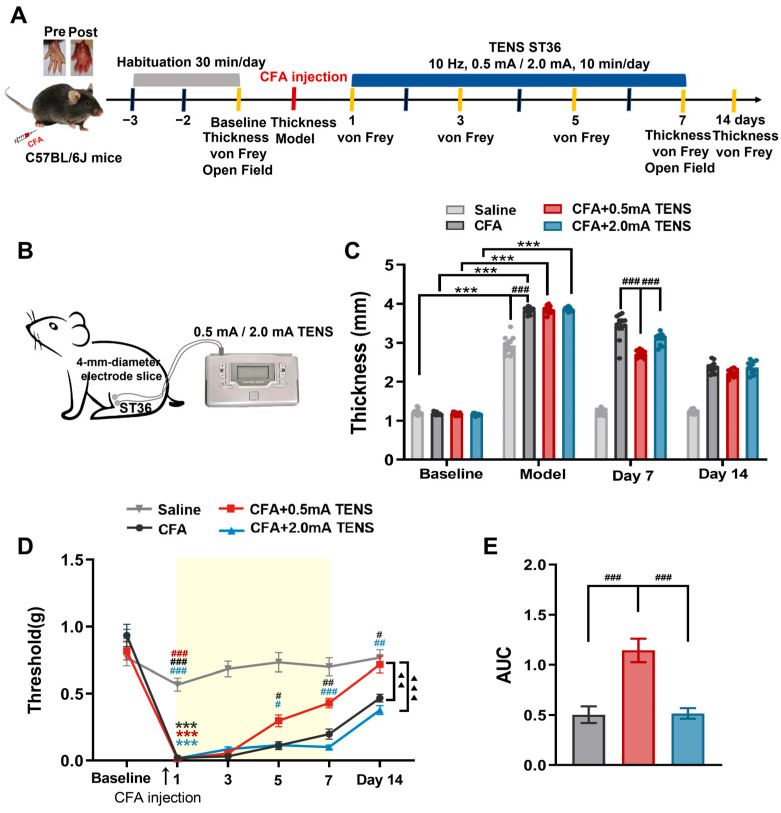
Effects of TENS ST36 on hind paw thickness and mechanical pain threshold in CFA mice. (**A**) Timeline of the CFA injection, transcutaneous electrical nerve stimulation (TENS), and behavioral testing to study the analgesic and anxiolytic effects of TENS (10 Hz, 0.5 mA/2.0 mA) treatment in CFA mice. The gray shading indicates the habituation period, the blue bar indicates the TENS stimulation period, and the red dashed line indicates the time point of CFA injection. (**B**) Schematic of TENS at the ST36 sites. (**C**) Time course of TENS on hind paw thickness of CFA mice (*n*= 12). (**D**) Time course of TENS on mechanical pain thresholds of CFA mice (*n* = 12). Yellow shadow is used for TENSs. (**E**) The AUC statistics of each group. All data are shown as mean ± S.E.M. (**C**,**D**) Two-way repeated measures ANOVA with Bonferroni post hoc test. Statistical symbols in different colors are used to denote different groups. Compared with baseline, *** *p* < 0.001; compared between groups at each time point, ^#^ *p* < 0.05, ^##^ *p* < 0.01, ^###^ *p* < 0.001; compared with CFA + 0.5 mA TENS group, ^▲▲^ *p* < 0.01, ^▲▲▲^ *p* < 0.001. (**E**) One-way repeated measures ANOVA with Bonferroni post hoc test, ^###^ *p* < 0.001.

**Figure 2:**
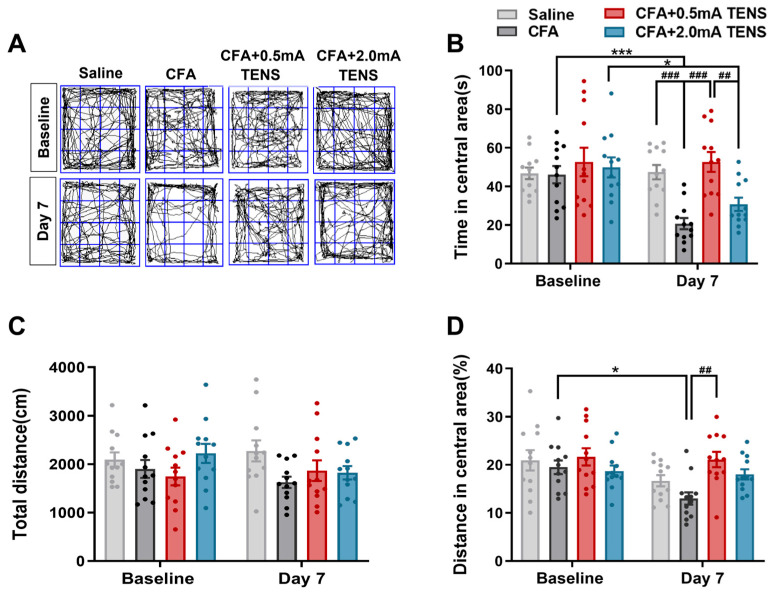
Effects of TENS ST36 on anxiety-like behaviors in CFA mice. (**A**) Representative animal tracks of the four groups in the open-field test. The blue square indicates the defined central area used for analysis. (**B**) The time in central area of mice in each group (*n* = 12). (**C**) The total distance of mice in each group (*n* = 12). (**D**) The proportion of the central area to the total distance in each group of mice (*n* = 12). All data are shown as mean ± S.E.M. (**B**–**D**) by two-way ANOVA with Bonferroni post hoc test. Compared within each group, * *p* < 0.05, *** *p* < 0.001; comparisons across all groups, ^##^ *p* < 0.01, ^###^ *p* < 0.001.

**Figure 3:**
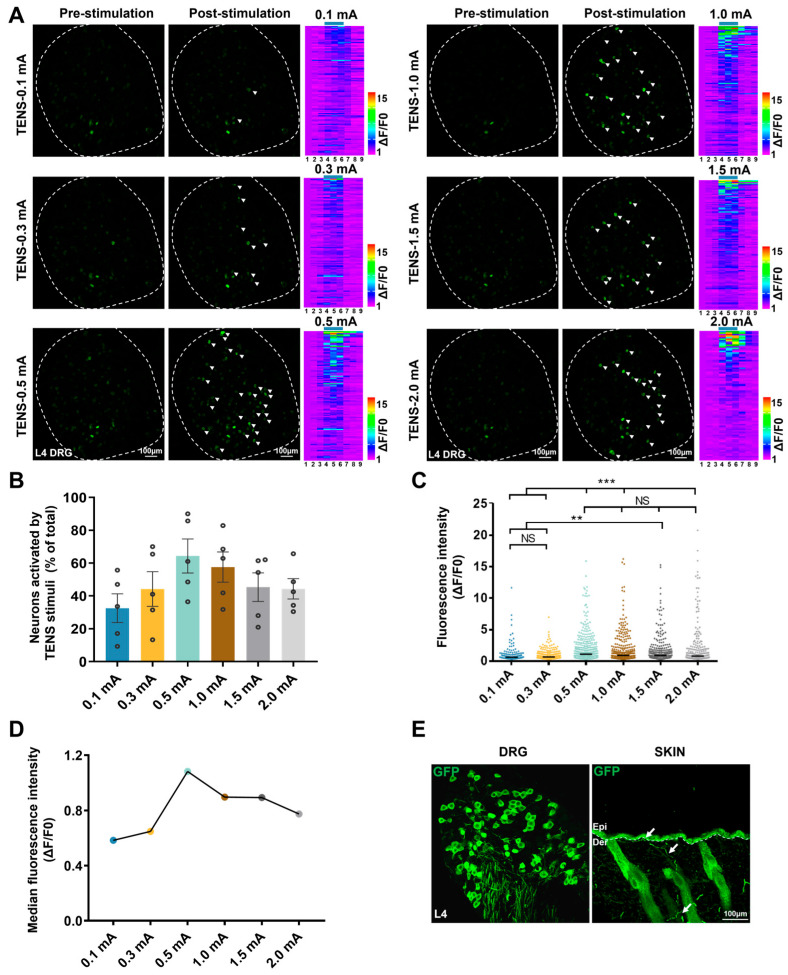
Responses of L4 DRG Mrgprb4-lineage neurons to diverse TENS in vivo Ca^2+^ imaging. (**A**) Representative images of Mrgprb4-lineage neuronal calcium transients to TENSs (0.1 mA, 0.3 mA, 0.5 mA, 1.0 mA, 1.5 mA, and 2.0 mA) observed during in vivo Ca^2+^ imaging of one L4 DRG (the white outline indicates the DRG border). Examples of activated neurons are marked by white arrowheads. (**Right**) Heatmaps of calcium signals in a single mouse DRG under diverse TENSs (total recorded cells = 200). The numbers of Mrgprb4-lineage neurons activated by TENS of 0.1 mA, 0.3 mA, 0.5 mA, 1.0 mA, 1.5 mA, and 2.0 mA were 94, 82, 122, 137, 124, and 85, respectively. Scale bar, 100 μm. (**B**) The proportion of Mrgprb4-lineage neurons activated by TENS at varying intensities. Each pair of open circles represents an individual mouse (*n* = 5). (**C**) Quantification of Ca^2+^ responses in cells responding to diverse TENSs. Violin plots show median (black lines) and data distributions. *n* = 5 (total recorded cells = 622). (**D**) Median fluorescence intensity of Mrgprb4-lineage neurons in response to TENS at varying intensities. (**E**) Representative images of GFP^+^ (green) cells in the Mrgprb4^Cre^; Rosa^ChR2-EYFP^ mice. Immunohistochemistry was performed on L4 DRG and skin from Mrgprb4^Cre^; Rosa^ChR2-EYFP^ mice. White arrows indicate examples of GFP^+^ cells. Dashed lines indicate the boundary between the epidermis and dermis layers. Scale bar, 100 µm. All data are shown as mean ± S.E.M. (**B**,**C**) by one-way repeated measures ANOVA with Bonferroni post hoc test, data points (circles) represent individual neurons. NS, not significant; ** *p* < 0.01, *** *p* < 0.001.

**Figure 4:**
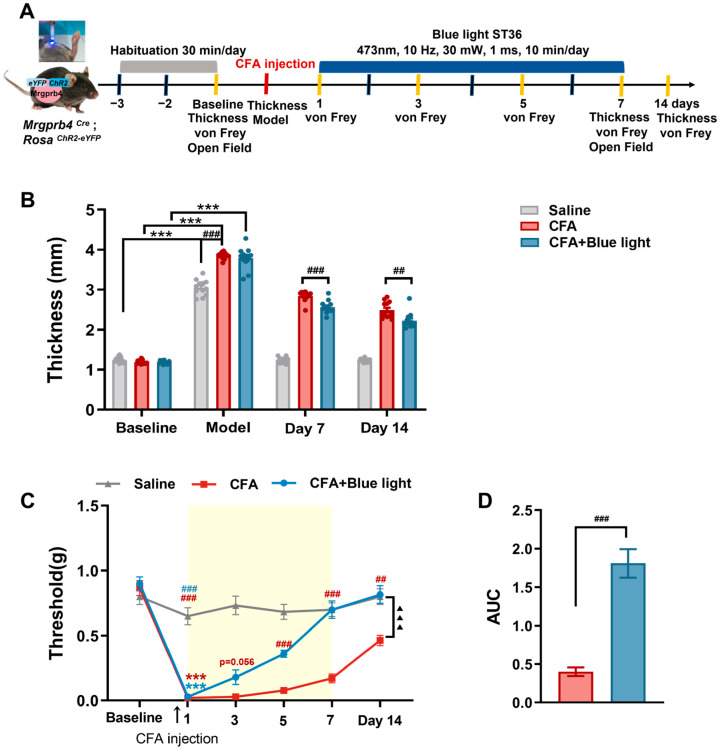
Effects of Mrgprb4-lineage neurons activated by blue light on hind paw thickness and mechanical pain threshold of CFA mice. The gray shading indicates the habituation period, the blue bar indicates the TENS stimulation period, and the red dashed line indicates the time point of CFA injection. (**A**) Timeline of the CFA injection, TENS, and behavioral testing to study the analgesic and anxiolytic effects of TENS (10 Hz, 0.5 mA/2.0 mA) treatment in CFA mice. (**B**) Time course of TENS on hind paw thickness of CFA mice (*n* = 12). (**C**) Time course of TENS on mechanical pain thresholds of CFA mice (*n* = 12). Yellow shadow is used for TENSs. (**D**) The AUC statistics of each group. All data are shown as mean ± S.E.M. (**B**,**C**) by two-way repeated measures ANOVA with Bonferroni post hoc test. Statistical symbols in different colors are represented to denote different groups. Compared with the baseline, *** *p* < 0.001; compared between groups at each time point, ^##^ *p* < 0.01, ^###^ *p* < 0.001; compared with CFA + 0.5 mA TENS group,^▲▲▲^ *p* < 0.001. (**D**) One-way repeated measures ANOVA with Bonferroni post hoc test, ^###^ *p* < 0.001.

**Figure 5:**
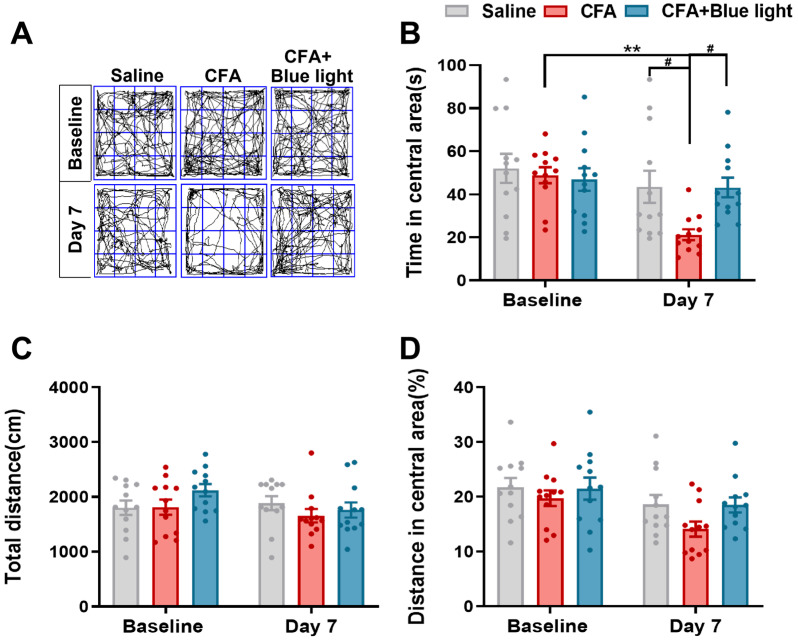
Effects of Mrgprb4-lineage neuron activated by blue light on anxiety-like behaviors of CFA mice. (**A**) Representative animal tracks of the three groups in the open-field test. The blue square indicates the defined central area used for analysis. (**B**) The time in central area of mice in each group (*n* = 12). (**C**) The total distance of mice in each group (*n* = 12). (**D**) The proportion of the central area to the total distance in each group of mice (*n* = 12). All data are shown as mean ± S.E.M. (**B**–**D**) Two-way ANOVA with Bonferroni post hoc test. Compared within each group, ** *p* < 0.01; comparisons across all groups, ^#^ *p* < 0.05.

**Figure 6:**
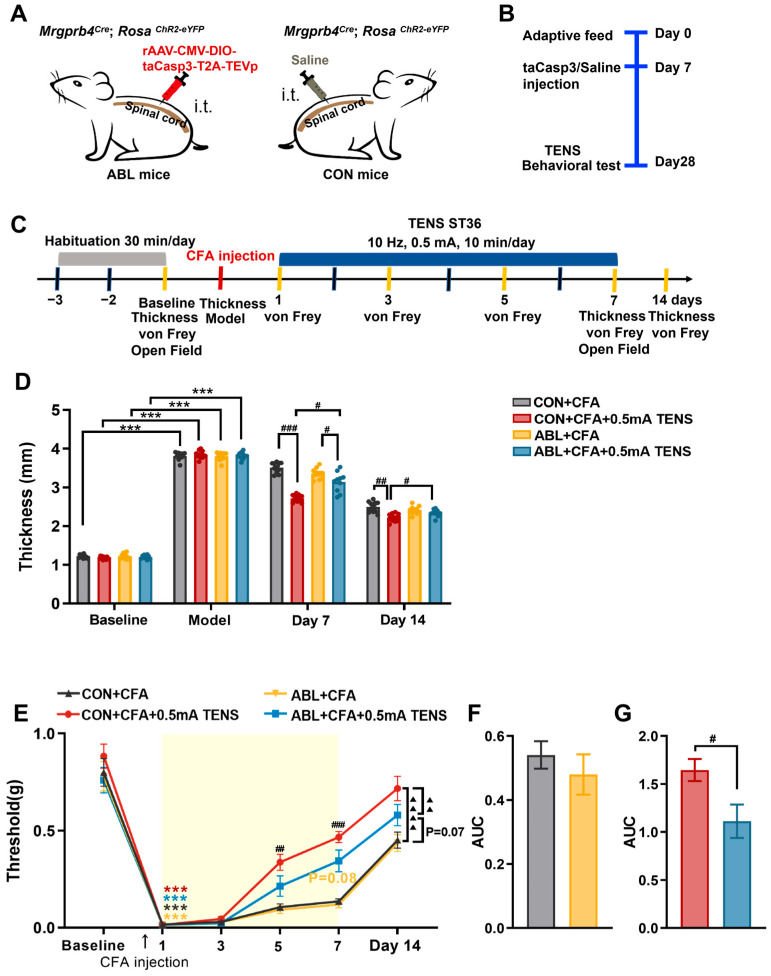
Effects of virus ablation of Mrgprb4-lineage neurons on hind paw thickness and mechanical pain threshold of CFA mice. (**A**) Diagram showing the Mrgprb4-lineage neurons’ virus ablation strategy. ChR2-eYFP is expressed in Mrgprb4-lineage neurons. Intrathecal injection with rAAV-CMV-DIO-taCasp3-T2A-TEVp (red) or saline in Mrgprb4^Cre^; Rosa*^ChR2-eYFP^* mice. (**B**) Diagram showing the experimental procedure. Adaptive feed on day 0, injection of taCasp3 or saline on day 7, and TENS or behavioral test on day 28. (**C**) Timeline of the CFA injection, TENS, and behavioral testing to study the analgesic and anxiolytic effects of TENS (10 Hz, 0.5 mA) treatment in CFA mice. The gray shading indicates the habituation period, the blue bar indicates the TENS stimulation period, and the red dashed line indicates the time point of CFA injection. (**D**) Time course of TENS on hind paw thickness of CFA mice (*n* = 10–12). (**E**) Time course of TENS on mechanical pain thresholds of CFA mice (*n* = 10–12). Yellow shadow is used for TENSs. (**F**,**G**) The AUC statistics of each group. All data are shown as mean ± S.E.M. (**D**,**E**) Two-way repeated measures ANOVA with Bonferroni post hoc test. Statistical symbols in different colors are used to denote different groups. Compared with baseline, *** *p* < 0.001; compared between groups at each time point, ^#^ *p* < 0.05, ^##^ *p* < 0.01, ^###^ *p* < 0.001; comparisons across all groups, ^▲▲^ *p* < 0.01, ^▲▲▲▲^ *p* < 0.0001. (**F**,**G**) Unpaired *t* test, ^#^ *p* < 0.05.

**Figure 7:**
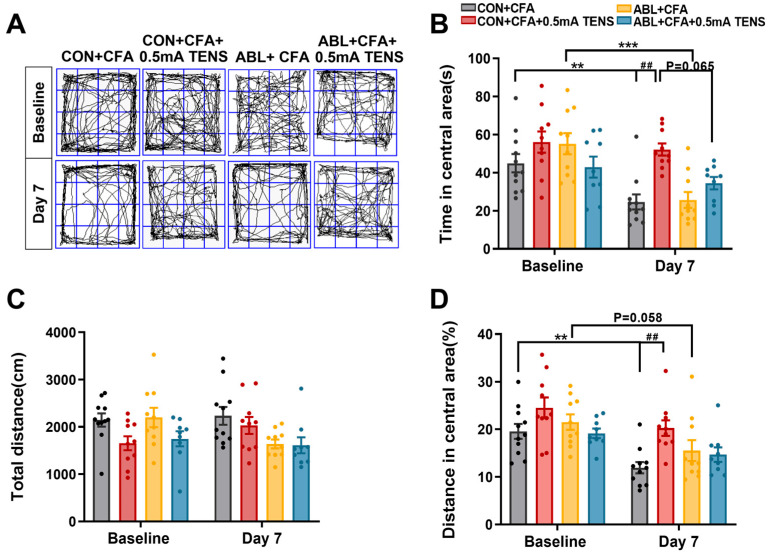
Effects of virus ablation of Mrgprb4-lineage neurons on anxiety-like behaviors in CFA mice. (**A**) Representative animal tracks of the four groups in the open-field test. The blue square indicates the defined central area used for analysis. (**B**) The time in central area of mice in each group (*n* = 10–12). (**C**) The total distance of mice in each group (*n* = 10–12). (**D**) The proportion of the central area to the total distance in each group of mice (*n* = 10–12). All data are shown as mean ± S.E.M. (**B**–**D**) Two-way ANOVA with Bonferroni post hoc test. Compared within each group, ** *p* < 0.01, *** *p* < 0.001; comparisons across all groups, ^##^ *p* < 0.01.

The mechanical threshold was assessed using sequentially ascending calibrated von Frey filaments (0.008 g, 0.02 g, 0.04 g, 0.07 g, 0.16 g, 0.4 g, 0.6 g, 1.0 g, 1.4 g; (Touch Test^®^ Sensory Evaluators, Shanghai RuiShi, Shanghai, China), commencing with the 0.008 g filament. Filaments were applied perpendicularly to the skin with sufficient force to produce slight bending, only during periods of mouse stillness. Positive responses were defined as paw withdrawal, paw licking, or escape behavior. Each filament underwent five consecutive applications; the minimal filament eliciting reflexive paw withdrawal on at least three of five trials was designated the paw withdrawal threshold, consistent with established methodology [29]. Sample sizes for each experiment were as follows: C57BL/6J CFA experiments (Figure 1 and Figure 2), *n* = 12 mice per group; in vivo Ca^2+^ imaging (Figure 3), *n* = 5 mice; Mrgprb4-ChR2 optogenetic experiments (Figure 4 and Figure 5), *n*= 12 mice per group; viral ablation experiments (Figure 6 and Figure 7), *n* = 10–12 mice per group. Within each experiment, the same cohort of animals was used for both the mechanical pain threshold assessments (days 1, 3, 5, 7, and 14) and the open-field test (day −1 and 7); no animals were designated exclusively for a single behavioral test.

### 2.5. Transcutaneous Electrical Nerve Stimulation Applications

The ST36 point was identified using an experimental acupuncture atlas [30]. Located at the posterolateral aspect of the knee joint, approximately 5 mm below the fibular head, ST36 served as the target site. Mice received induction anesthesia with 2% isoflurane, followed by maintenance at 1% concentration. Positioned supine on the operating table, each mouse had a 4 mm diameter silver wire electrode secured over the right ST36 point. TENS intervention commenced on the first post-modeling day. The electrode, connected to a HANS-200A electroacupuncture instrument, delivered TENS as depicted in Figure 1B. A sufficient quantity of conductive paste coated the electrode slice surface to ensure optimal conductivity. Stimulation parameters were 10 Hz frequency and 0.5 mA/2.0 mA intensity, administered for 10 min per session, once daily, over 7 consecutive days.

### 2.6. Open-Field Test

Animals were acclimated to both their testing environment and equipment before behavioral assessments commenced. The open-field test was performed on the same day as the mechanical pain threshold assessment (day −1 baseline and day 7 post-TENS), using the same cohort of animals. To minimize potential order effects, the von Frey test was conducted first, followed by the open-field test at least 1 h later, with both tests completed in the morning session. Throughout testing, the experimenter remained blind to each animal’s genotype until after behavioral analysis concluded. Stress and anxiety-like behaviors were quantified using an open-field apparatus (L40 × W40 × H30 cm) [29]. Following a 10 min adaptation period within the experimental room, each mouse was gently placed in the center of the open-field arena. The apparatus floor was divided into 16 equal squares, designating the middle four squares as the center zone. A digital video camera (Canon, Japan) recorded each animal’s movements for 5 min, specifically tracking the total distance traveled and time spent within the center zone. The mice’s motion trails were subsequently analyzed using Any-maze software (version 7, Stoelting).

### 2.7. In Vivo Ca^2+^ Imaging of L4 DRG

As previously described [12], endotracheal intubation and exposure of the L4 DRG were performed (Figure S1A–C). Intracellular calcium concentration shifts were captured using a laser-scanning confocal microscope (Leica Stellaris 8, Wetzlar, Germany), with changes visualized through fluctuating fluorescein intensity within the neurons. Before imaging, mice were secured prone on a custom-designed microscope stage. The spinal column was firmly stabilized using a pair of customized spinal clamps to eliminate movement artifacts (Figure S1D) and guarantee the L4 DRG remained fully exposed, free from blood exudation obscuring the field, consistent with prior methodology [31,32,33]. During imaging sessions, adult male or female Mrgprb4^Cre^; GCaMP6s mice received anesthesia via tracheal intubation (0.5–1% isoflurane, R510-22-10, RWD) following L4 DRG exposure. Supplemental 0.5% isoflurane was administered intratracheally when necessary to prevent muscle twitching during recording if anesthesia proved to be insufficient. Ointment was applied to the animals’ eyes to prevent drying.

Imaging was conducted using a Leica 10× air objective at 1× magnification. Following localization of the dorsal root ganglion (DRG) field under microscopy (Figure S1E), L4 DRG thickness was determined along the Z-axis, with subsequent adjustment of X and Y planes to ensure complete inclusion of the L4 DRG within the imaging field. Time-lapse z-stacks of intact DRG were acquired at 512 × 512 resolution. Individual frames comprised 6–12 z-stacks (contingent upon DRG-to-objective lens alignment), with 9 frames captured for mechanical stimuli and 20 frames for thermal stimuli. A 488 nm excitation wavelength was employed at 5% laser power, with bidirectional image acquisition at 400 Hz scan speed. Neuronal activation triggers GCaMP binding to intracellular Ca^2+^, yielding green fluorescence for imaging (Figure S1F). Live imaging spanned 9 consecutive frames: frames 1–3 recorded baseline fluorescence intensity (pre-stimulation), frames 4–6 documented stimulation-phase intensity, and frames 7–9 captured post-stimulation recovery.

Transcutaneous electrical nerve stimulation (TENS) was then administered at randomized intensities (0.1, 0.3, 0.5, 1.0, 1.5, 2.0 mA; 10 Hz, 1 ms pulse width) at ST36, with 1–2 min interstimulus intervals. Core body temperature was maintained at 37.0 ± 0.5 °C via heating pad with rectal thermometry (DC Temperature Controller, FSH, Chattahoochee, FL, USA), while respiratory parameters underwent real-time monitoring (small animal anesthesia system, SomnoSuite, Kent Scientific, Torrington, CT, USA).

### 2.8. Optogenetics

The hair in the right ST36 area of the mice was removed in advance. Under maintained isoflurane inhalation anesthesia, the right ST36 area was subjected to blue light stimulus. The optical fiber was positioned 5–7 mm above the skin surface. Parameters: 473 nm, 10 Hz, 30 mW, 1 ms, 10 min per time, once a day, for 7 consecutive days. The body temperature of the mice was maintained at 37.0 ± 0.5 °C with a heating pad.

### 2.9. Immunofluorescence

Animals were deeply anesthetized with a tribromoethanol solution, and the blood was cleared from all tissues by perfusing saline through the vascular system. Mice were then perfusion-fixed using 4% paraformaldehyde (PFA). Tissues were then collected and post-fixed in 4% PFA accordingly (DRG: 2 h, skin: 2–3 h). All tissues were cryoprotected in 30% sucrose for a minimum of 48 h. Subsequently, the tissues were embedded and sectioned on a freezing microtome (LEICA CM 1950, Wetzlar, Germany) (DRG: 20 μm, skin: 30 μm). Sections were washed in PBS (3 × 10 min) and then blocked in PBS containing 3% goat serum and 0.5% Triton X-100 for 1 h at room temperature. Chicken anti-GFP (1:500, Abcam, #ab13970) was used as the primary antibody. Anti-GFP antibodies were used to label the expression of GCaMP6s in Mrgprb4-GCaMP6s mice or ChR2 in Mrgprb4-ChR2 mice. The secondary antibody was goat anti-chicken IgG-Alexa-Fluor 488 (1:600, Invitrogen, A11039). DAPI-containing media (ZLI-9600, ZSGB-BIO) or glycerin was used to coverslip the tissue. The DRG sections were imaged with full-spectral scanning for confocal microscopy (OLYMPUS FV1200, Tokyo, Japan).

### 2.10. Quantification of Calcium Imaging

Calcium imaging data analysis was performed by Image J (1.52p, National Institutes of Health). After importing the collected raw data into Image J, the activated neurons were manually circled, and the relative fluorescence intensity of the neurons was exported. The calcium signal transients are expressed as ΔF/F0 = (Ft − F0)/F0. Ft represented the maximum fluorescence intensity of cells during stimulation (4–6 frames), and F0 represented the maximum fluorescence intensity of cells at baseline (1–3 frames) before intervention. Activation in neurons was defined as an increase in ΔF/F0 ≥ 30% [31]. Calcium imaging data processors were mutually blinded to the experimental operator to reduce selection and bias.

### 2.11. Statistical Analysis

All data were presented as mean ± standard error of the mean (S.E.M). Statistical analyses were performed using GraphPad Prism 8.0 software (GraphPad Software, Inc., La Jolla, CA, USA). Figures were prepared with bioRender (https://BioRender.com), GraphPad Prism 8.0. Two-tailed unpaired or paired *t* tests were used to compare the two groups. Multiple groups were compared using one-way ANOVA or two-way ANOVA followed by Bonferroni post hoc tests. Data that did not conform to normal distribution were analyzed using non-parametric tests. The number of mice and the statistical tests used for individual experiments were included in the Fig. legends. *p* < 0.05 was considered statistically significant in all tests.

## 3. Results

### 3.1. Effects of TENS ST36 on Hind Paw Thickness and Mechanical Pain Threshold in CFA Mice

The hind paw thickness and mechanical pain threshold of mice in each group were observed, as shown in Figure 1. TENS was applied to the right ST36 acupoint using a HANS-200A (Figure 1A,B). There was no significant difference in the baseline between the groups. The paw swelling of mice in each group was obvious after CFA injection (Figure 1C). Moreover, mechanical hyperalgesia was observed in CFA mice, and the mechanical pain threshold decreased significantly (Figure 1D).

After 7 days of TENS intervention, compared with the CFA group, the paw thickness in the 0.5 mA TENS group decreased significantly (*p* = 0.0002), while that in the 2.0 mA TENS group did not show a significant decrease (Figure 1C). There was no statistically significant difference in paw thickness between the CFA group and the TENS group on day 14.

When comparing within each time-point group, the mechanical pain threshold of mice in each group showed no significant change after 1 and 3 days of TENS intervention (Figure 1D). Compared with the CFA group and the 2.0 mA TENS group, the mechanical pain threshold in the 0.5 mA TENS group increased on day 5 (*p* = 0.0444, *p* = 0.0173) and showed a significant increase on day 7 (*p* = 0.0001, *p* < 0.0001). After completion of the intervention, compared to the CFA group and the 2.0 mA TENS group, the mechanical pain threshold in the 0.5 mA TENS group was significantly higher on day 14 (*p* = 0.0267, *p* = 0.0065). Global comparisons between groups demonstrated that the mechanical pain threshold in the 0.5 mA TENS group was significantly higher compared to the CFA and 2.0 mA TENS groups (Figure 1D, *p* < 0.001).

Furthermore, the area under the curve (AUC) was employed to visually assess the impact of TENS on mechanical pain thresholds. As shown in Figure 1E, the analgesic effect was significantly more pronounced in the 0.5 mA TENS group compared with both the CFA group and the 2.0 mA TENS group (*p* < 0.001). These data suggest that 0.5 mA TENS can reduce paw thickness and elevate mechanical pain thresholds in CFA mice, exhibiting a superior efficacy to that of 2.0 mA TENS.

### 3.2. Effects of TENS ST36 on Anxiety-like Behaviors in CFA Mice

To assess anxiety-like behavior in mice, we employed the open-field test. As illustrated in Figure 2, baseline measurements showed no significant differences across all groups. By day 7, mice in the CFA group exhibited pronounced anxiety-like behavior, demonstrating a statistically significant reduction in both time spent within the central area and the proportion of central area distance traveled relative to their baseline (Figure 2A,B,D). The 2.0 mA TENS group also showed a significant decrease in central area time, while the 0.5 mA TENS group showed no significant change. Intergroup comparisons revealed that the CFA group spent significantly less time in the central area than the saline group (Figure 2B, *p* = 0.0006). Notably, the 0.5 mA TENS group showed a significant increase in both central area time and the central area distance proportion compared to the CFA group (Figure 2B, *p* < 0.0001; Figure 2D, *p* = 0.0013). Furthermore, the 0.5 mA TENS group spent significantly more time in the central area than the 2.0 mA TENS group (*p* = 0.0072). Total distance traveled remained consistent across all groups (Figure 2C). Collectively, these findings demonstrate that 0.5 mA TENS effectively alleviates CFA-induced anxiety-like behaviors in mice.

### 3.3. Responses of L4 DRG Mrgprb4-Lineage Neurons to Diverse TENS in Vivo Ca^2+^ Imaging

To investigate the responses of Mrgprb4-lineage neurons to varying TENS intensities, *Mrgprb4*-GCaMP6s mice underwent in vivo Ca^2+^ imaging of L4 DRG (Figure S2A). The flow chart depicting in vivo imaging and TENS at ST36 appears in Figure S2B. Briefly, TENS activated Mrgprb4-lineage neurons across all tested intensities, with no significant difference in the response ratio (Figure 3A,B). Compared to 0.1 mA and 0.3 mA TENS, markedly higher fluorescence intensity was observed with 0.5 mA, 1.0 mA, 1.5 mA, and 2.0 mA TENS (Figure 3C). Furthermore, fluorescence intensity progressively increased from 0.1 mA to 0.5 mA TENS but gradually decreased from 0.5 mA to 2.0 mA TENS (Figure 3D). Therefore, 0.5 mA emerged as the optimal intensity for activating Mrgprb4-lineage neurons. Post-experiment, L4 DRG and skin samples were harvested for immunofluorescence staining. As Figure 3E illustrates, Mrgprb4-lineage neurons and positive neurons and/or fibers were observed in both the L4 DRG and the hairy skin, confirming the successful establishment of the *Mrgprb4*-GCaMP6s mouse model.

### 3.4. Effects of Mrgprb4-Lineage Neurons Activated by Blue Light on Hind Paw Thickness and Mechanical Pain Threshold of CFA Mice

Healthy Mrgprb4^Cre^; Rosa^ChR2-eYFP^ mice underwent a 3-day adaptation period, followed by a subcutaneous injection of 25 μL CFA into their right plantar. ChR2-eYFP expression was specific to Mrgprb4-lineage neurons (Figure S3C). To simulate TENS, blue light (473 nm, 30 mW, 10 Hz, 10 min/day) irradiated the ST36 acupoint. Mechanical pain thresholds were assessed on days 1, 3, 5, and 7 post-light exposure (Figure 4A). Baseline thresholds showed no significant difference between groups, and paw swelling was evident in all mice after modeling (Figure 4B). Intraplantar CFA injection induced mechanical hyperalgesia, significantly lowering the mechanical pain threshold, whereas saline injection caused no significant change (Figure 4C). Following seven days of blue light intervention, paw thickness significantly decreased in the blue light group compared to the CFA group (Figure 4B, *p* = 0.0003); however, by day 14, paw thickness in the blue light group significantly increased relative to the CFA group (Figure 4B, *p* = 0.0119). Within time points, the blue light group exhibited a trend towards an elevated mechanical pain threshold on day 3 (*p* = 0.056) and significant increases on days 5 and 7 compared to the CFA group (Figure 4C, *p* < 0.0001, *p* = 0.0027). Post-intervention, the blue light group also showed a significantly increased mechanical pain threshold on day 14 compared to the CFA group. Overall, global comparisons confirmed that the mechanical pain threshold was significantly higher in the blue light group versus CFA groups (Figure 4C, *p* < 0.0001). Furthermore, AUC analysis visually demonstrated blue light’s significant analgesic effect relative to the CFA group (Figure 4D, *p* < 0.0001). These findings indicate that blue light activation of Mrgprb4 lineage neurons reduces paw thickness and elevates mechanical pain thresholds in CFA-treated mice.

### 3.5. Effects of Mrgprb4-Lineage Neurons Activated by Blue Light on Anxiety-like Behaviors of CFA Mice

As shown in Figure 5, there was no significant difference in the baseline among all groups. On day 7, compared with the baseline, the CFA group exhibited anxiety-like behaviors, with a statistically significant decrease in the time spent in the central area (Figure 5A,B). Comparison among groups showed that time in central area in the CFA group was significantly reduced compared with the saline group (Figure 5B, *p* = 0.0350). Compared with the CFA group, the time in central area of the blue light group was significantly increased (Figure 5B, *p* = 0.0380). There was no significant difference observed in either the total distance of motion or the proportion of the central area to the total distance (Figure 5C,D). These data indicate that blue light could relieve the anxiety-like behaviors associated with CFA mice.

### 3.6. Effects of Virus Ablation of Mrgprb4-Lineage Neurons on Hind Paw Thickness and Mechanical Pain Threshold of CFA Mice

To evaluate the impact of DRG Mrgprb4-lineage neurons on mechanical pain thresholds and anxiety-like behaviors, after seven days of adaptive feeding, we first generated Mrgprb4^Cre^; Rosa^ChR2-EYFP^ mice. This involved intrathecal injection of a Cre-dependent taCasp3 virus to ablate Mrgprb4-lineage neurons, creating ablation (ABL) mice (Figure 6A). Littermate control (CON) mice received an equivalent volume of 0.9% saline (Figure 6A). Subsequently, TENS and behavioral testing commenced in both ABL and CON mice at least 21 days post-intrathecal surgery (Figure 6B). Immunofluorescence staining assessed the expression of Mrgprb4-lineage neurons and fibers within the L3-L5 DRGs and ST36 regions of these mice. Strikingly, immunofluorescence revealed a significant reduction in Mrgprb4-lineage neurons within the L3-L5 DRGs of ABL mice compared to CON mice. Ablation efficiency reached 45.97%, 47.55%, and 44.27% (Figure S3A,B). Furthermore, Mrgprb4-positive fiber density in the ST36 region skin of ABL mice appeared strikingly low (Figure S3C). These results confirm the successful generation of the ABL mouse.

The effects of 0.5 mA TENS applied at ST36 on paw thickness and mechanical pain threshold in ABL and CON mice are depicted in Figure 6C-E. Mechanical pain thresholds were assessed on days 1, 3, 5, and 7 post-TENS administration (Figure 6C). Baseline measurements revealed no significant intergroup differences (Figure 6D,E). Following modeling, all groups developed marked paw swelling (Figure 6D) and exhibited significantly reduced mechanical pain thresholds (Figure 6E). After 7 days of TENS intervention, the CON + CFA + 0.5 mA TENS group showed significantly diminished paw thickness compared to the CON + CFA group (Figure 6D, *p* < 0.0001). Similarly, the ABL + CFA + 0.5 mA TENS group demonstrated substantially lower paw thickness than the ABL + CFA group (Figure 6D, *p* = 0.0225). However, the ABL + CFA + 0.5 mA TENS group displayed significantly greater hind paw swelling than the CON + CFA + 0.5 mA TENS group (Figure 6D, *p* = 0.0137). By day 14, the CON + CFA + 0.5 mA TENS group maintained significantly reduced paw thickness relative to the CON + CFA group (Figure 6D, *p* = 0.0040). Concurrently, the ABL + CFA + 0.5 mA TENS group continued to exhibit significantly more pronounced swelling than the CON + CFA + 0.5 mA TENS group (Figure 6D, *p* = 0.0187). Compared within each time-point group, the mechanical pain threshold of mice in each group had no significant change after day 1 and 3 of intervention (Figure 6E). Compared with the CON + CFA group, the mechanical pain threshold in the CON + CFA + 0.5 mA TENS group was significantly increased on day 5 and 7 of intervention (Figure 6E, *p* = 0.0012, *p* > 0.0001). Compared with the ABL + CFA group, the mechanical pain threshold of the ABL + CFA + 0.5 mA TENS group had no significant change on day 5, whereas an increasing trend was observed on day 7 (Figure 6E, *p* = 0.08). After the completion of the intervention, there was no significant change in mechanical pain threshold on day 14 (Figure 6E). Global comparisons between groups demonstrated that the mechanical pain threshold in the CON + CFA + 0.5 mA TENS group was significantly increased compared to the CON + CFA group (Figure 6E, *p* < 0.0001). Compared with the ABL + CFA group, the mechanical pain threshold of the ABL + CFA + 0.5 mA TENS group tended to be increased (Figure 6E, *p* = 0.07). Compared with the CON + CFA + 0.5 mA TENS group, the mechanical pain threshold of the ABL + CFA + 0.5 mA TENS group was significantly decreased (Figure 6E, *p* = 0.0021). Furthermore, AUC was employed to visually evaluate the impact of Mrgprb4-lineage neuron ablation on mechanical pain thresholds as shown in Figure 6F,G. There was no statistically significant difference in the AUC between the CON + CFA group and the ABL + CFA group, indicating that ablation of Mrgprb4-lineage neurons had no substantial impact on the CFA model (Figure 6F). Compared with the CON + CFA + 0.5 mA TENS group, the analgesic effect was significantly attenuated in the ABL + CFA + 0.5 mA TENS group (Figure 6G, *p* = 0.0159). These data suggest that the analgesic efficacy of 0.5 mA TENS is compromised following the ablation of Mrgprb4-lineage neurons.

### 3.7. Effects of Virus Ablation of Mrgprb4-Lineage Neurons on Anxiety-like Behaviors in CFA Mice

As shown in Figure 7, the baseline measurements revealed no significant differences among all groups. By day 7, relative to the baseline, the CON + CFA group demonstrated pronounced anxiety-like behaviors, evidenced by a statistically significant decrease in both the time spent in the central area and the proportion of central area to total distance (Figure 7A,B,D). In contrast, the ABL + CFA group showed a significant decrease only in central area time, while the proportion of central area distance exhibited a tendency to increase (Figure 7A,B,D). Compared to the CON + CFA group, the CON + CFA + 0.5 mA TENS group displayed a significantly increased central area residence time (*p* = 0.0004). Furthermore, relative to the ABL + CFA group, the ABL + CFA + 0.5 mA TENS group exhibited a tendency towards increased central area time (Figure 7B, *p* = 0.058). However, the central area time in the ABL + CFA + 0.5 mA TENS group was not significantly increased compared to the CON + CFA + 0.5 mA TENS group. Compared to the CON + CFA group, the proportion of central area distance to total distance was significantly increased in the CON + CFA + 0.5 mA TENS group (Figure 7D, *p* = 0.0043). Importantly, the total distance traveled showed no statistical differences across any groups (Figure 7C). Collectively, these findings indicate that viral ablation of Mrgprb4-lineage neurons abolished the ability of TENS to alleviate anxiety-like behaviors.

## 4. Discussion

This study employed transgenic mice and a CFA-induced model of chronic pain and anxiety comorbidity. Utilizing in vivo Ca^2+^ imaging, genetic manipulation, viral strategies, and behavioral assessments, our research demonstrated that 0.5 mA TENS applied to ST36 significantly ameliorated both pain and anxiety-like behaviors in the comorbidity model. Simultaneously, in vivo Ca^2+^ imaging confirmed that a substantial proportion of Mrgprb4-lineage neurons were activated by this specific TENS intensity. Photostimulation targeting ST36 replicated these analgesic and anxiolytic effects by selectively activating Mrgprb4-lineage neurons. Conversely, viral ablation of these neurons markedly attenuated the analgesic benefits of 0.5 mA TENS and failed to alleviate anxiety-like behaviors. Collectively, these findings indicate that the therapeutic regulation of chronic pain and anxiety comorbidity by 0.5 mA TENS critically requires the functional involvement of Mrgprb4-lineage neurons.

TENS is a non-invasive therapy that delivers electrical currents through skin electrodes to stimulate the nervous system [34,35]. The specific fiber types activated depend on stimulation frequency and intensity [36,37,38,39]. Low-frequency TENS primarily activates C fibers [38], including Mrgprb4-lineage C-low-threshold mechanoreceptors, whereas high-frequency TENS preferentially engages Aβ fibers [39,40]. TENS has demonstrated efficacy in alleviating chronic pain conditions such as inflammatory pain and fibromyalgia [17,18,20,21,22]. Furthermore, 0.5 mA EA demonstrates superior efficacy in alleviating inflammatory edema and hyperalgesia [41] and has shown potential for treating anxiety disorders [24,25,26]. The ST36 acupoint, targeted in this study, is commonly used for managing pain and emotional disorders [27,42]. These features support the clinical potential of TENS for chronic pain comorbid with anxiety.

The CFA-induced inflammatory pain model is the most commonly used animal model for studying comorbidity of chronic pain and anxiety [43,44]. This model can induce persistent mechanical pain sensitivity behavior by subcutaneous injection of CFA into the hind paw of mice [45]. Studies have shown that mice injected with CFA exhibited anxiety-like behaviors on the 7th day, primarily manifested as a notable reduction in the time spent in the central area during an open-field test [46].

The analgesic effects of TENS may be mediated through the gate control theory or the endogenous analgesic system. According to the classical gate control theory, excitation of large-diameter afferent fibers prompts substantia gelatinosa cells in the spinal dorsal horn to release inhibitory neurotransmitters that close the “gate” via presynaptic inhibition of T cells, whereas excitation of small-diameter afferent fibers (thin fibers) opens the “gate” and elicits pain [47]. TENS is generally believed to produce its segmental analgesic effect by activating thick fibers to inhibit thin-fiber-mediated nociceptive transmission [38]. However, spinal pain modulation is far more complex than the traditional gate control theory describes. Lu and Perl et al. discovered that low-threshold C-fibers regulate nociceptive C-fibers input through inhibitory interneurons, demonstrating that C-fibers are also capable of closing the pain “gate” [48]. Using in vivo Ca^2+^ imaging, we confirmed that Mrgprb4-lineage neurons are polymodal, encompassing both C-low-threshold mechanoreceptors and C-high-threshold mechanoreceptors [12]. As shown in Figure 3, Mrgprb4-lineage neurons innervating hairy skin are activated by TENS, with 0.5 mA emerging as the optimal stimulation intensity. Accordingly, 0.5 mA TENS at ST36 effectively closes the pain “gate” by engaging Mrgprb4-lineage fiber terminals in the skin. The selection of 0.5 mA over 2.0 mA reflects a fundamental principle of fiber recruitment: at higher intensities, Ca^2+^ imaging data showed that Mrgprb4-lineage neuron activation did not reach the proportion required for analgesia (Figure 3), consistent with a shift in the fiber recruitment profile toward pro-nociceptive pathways that counteracts the analgesic benefit.

Dopamine (DA) plays a crucial role in reward processing, pain modulation, and the pathophysiology of affective disorders, and reduced DA synthesis or dysfunction can lead to anxiety-like behaviors [49]. Mrgprb4-lineage neurons form synaptic connections with G protein-coupled receptor 83 (Gpr83)-expressing spinoparabrachial (SPB) neurons [14,50]. Optogenetic activation of Mrgprb4-lineage neurons conveys sensory information via Gpr83^+^ SPB neurons to the parabrachial nucleus (PBN) and ultimately to the nucleus accumbens (NAc), triggering DA release and producing conditioned place preference, indicating positive reinforcing and anxiolytic effects [14,51,52]. Our results demonstrate that ablating Mrgprb4-lineage neurons significantly attenuated the anxiolytic effects of 0.5 mA TENS (Figure 7), confirming that these neurons are required for TENS-mediated anxiety relief.

The peripheral-to-central mechanistic axis engaged by TENS at ST36 is supported by convergent evidence from circuit-level and neuroproteomics evidence. We propose that peripheral activation of Mrgprb4-lineage DRG neurons is transduced into supraspinal analgesia and anxiolysis via a hierarchically organized ascending circuit: at the spinal level, Mrgprb4-lineage DRG neurons relay signals to Gpr83^+^ SPB neurons, which project rostrally to the PBN—a key hub for nociceptive and interoceptive processing [50]; ascending PBN projections then reach mesolimbic structures, where the parabrachial–mesencephalic axis modulates ventral tegmental area (VTA) dopamine neuron activity [53,54]. This is further corroborated by proteomic evidence showing that peripheral nociceptive input induces coordinated neurochemical and neuroinflammatory alterations across a “nociceptive neuraxis” from peripheral nerve to spinal cord to orbitofrontal cortex, with DA-regulating proteins significantly upregulated in supraspinal affective regions [55]. Critically, the emotional valence of dopaminergic modulation within this axis is determined by projection-target specificity: VTA projections to the NAc promote positive valence and anxiolysis, whereas VTA projections to the interpeduncular nucleus drive anxiety-like states via distinct D1-receptor microcircuits [56,57]. The 0.5 mA TENS at ST36, by selectively recruiting Mrgprb4-lineage C-LTMRs, preferentially engages the NAc-projecting arm of the mesolimbic dopamine system, tilting dopaminergic tone toward anxiolysis—consistent with our ablation data (Figure 7) and prior optogenetic evidence [14]. Taken together, these findings suggest that Mrgprb4-lineage neurons serve as a dual-function peripheral node: at the spinal level, their activation closes the pain “gate” via inhibitory interneuron circuits to produce analgesia; simultaneously, ascending signals through the PBN–VTA–NAc axis engage mesolimbic dopaminergic tone to confer anxiolysis. This convergent peripheral-to-central architecture provides a mechanistic basis for the simultaneous amelioration of both pain and anxiety by 0.5 mA TENS at ST36. Nevertheless, direct central validation, including spinal dorsal horn activation markers, real-time NAc dopamine biosensing, and projection-selective chemogenetic manipulation of VTA terminals, was not performed in the present study and represents an important direction for future investigation.

Regarding clinical translation, the 0.5 mA intensity used in this mouse study requires contextualization before extrapolation to human TENS settings. Because murine skin impedance and electrode contact area differ substantially from human tissue, the critical variable is not absolute current but charge density per unit area and the resulting fiber recruitment profile. In human TENS targeting the ST36-equivalent region (tibialis anterior, fibular head vicinity), low-intensity settings that selectively engage large-diameter tactile afferents without recruiting nociceptors—typically 1–15 mA at low-to-moderate frequencies depending on electrode size and placement—may approximate the fiber-type selectivity achieved at 0.5 mA in mice. Future translational research should map the intensity–response relationship for C-LTMR activation in humans, using psychophysical pleasantness ratings or skin-nerve microneurography as surrogate measures, to establish evidence-based TENS dosing guidelines for comorbid chronic pain and anxiety.

## 5. Conclusions

**Figure 8:**
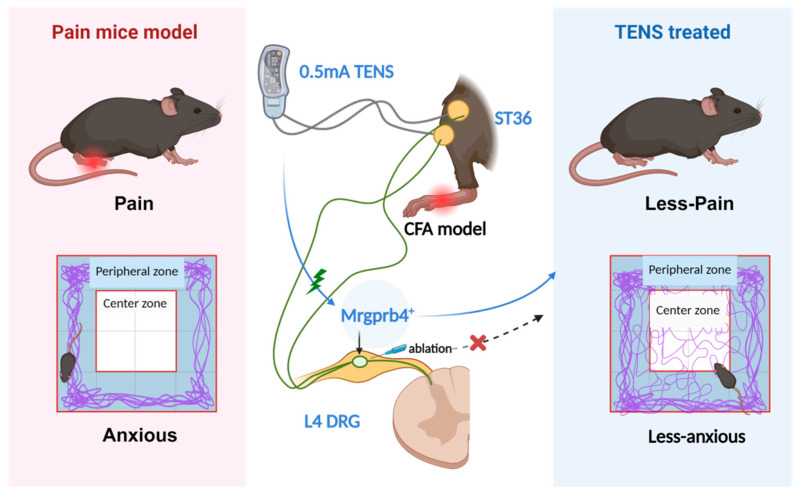
This schematic diagram depicts the role of Mrgprb4-lineage neurons in TENS-induced analgesia within a CFA-induced inflammatory pain model. Left panel: CFA-injected mice exhibit mechanical hyperalgesia and anxiety-like behavior in the open-field test. Central schematic: 0.5 mA TENS at ST36 preferentially activates Mrgprb4-lineage neurons in the L4 DRG, triggering ascending pathways (solid blue arrows) that produce coordinated analgesic and anxiolytic effects. Optogenetic activation (solid green arrows) of these neurons replicates both effects, while viral ablation (dashed arrow, red ✕) substantially attenuates them. Right panel: following TENS treatment, CFA mice show reduced hyperalgesia and restored center-zone exploration, indicating concurrent pain relief and anxiolysis. (created in BioRender. Cheng, H. (2026) https://BioRender.com/o04hmz0, accessed on 12 January 2026).

Based on compelling evidence that polymodal Mrgprb4-lineage neurons mediate pleasant sensation, this study further reveals these neurons mediate the regulatory effects of TENS at ST36 on comorbid chronic pain and anxiety (Figure 8). These results offer valuable novel insights and references for therapeutic targets addressing these co-occurring disorders and may significantly improve clinical efficacy.

### Limitations of the Study

This study establishes that the regulatory effect of TENS ST36 on chronic pain and anxiety comorbidity critically depends on polymodal Mrgprb4-lineage neurons. However, several limitations should be acknowledged. First, the absence of a sham TENS control group—in which electrodes would be placed over ST36 without current delivery—means that non-electrical procedural factors such as handling, immobilization, and anesthesia cannot be fully excluded as contributors to the observed therapeutic effects; future studies should incorporate this control to more rigorously isolate the contribution of electrical stimulation. Second, anxiety-like behavior was assessed using only the open-field test; although this assay is well validated in the CFA model and was prioritized to maintain consistent time points with pain assessments, future studies should include complementary paradigms such as the elevated plus maze or light–dark box. Third, neuronal activation was characterized exclusively at the DRG level, and spinal or supraspinal markers of central sensitization—such as c-Fos and pERK in the dorsal horn—were not assessed; direct in vivo validation of the proposed peripheral-to-central circuit axis therefore remains to be established. Fourth, although male and female mice were included in equal proportions across all groups, formal sex-stratified analyses were not performed; given the possibility of sex-specific differences in CFA-induced pain and anxiety-like behavior, future studies with prospectively powered sex-stratified designs are needed. Fifth, the effects of TENS at ST36 on thermal pain thresholds in CFA mice were not examined and warrant further investigation. Sixth, the temporal resolution of our in vivo calcium imaging paradigm is insufficient to capture millisecond-level neuronal encoding during TENS, representing an avenue for methodological refinement. Collectively, future research should further elucidate the intrinsic mechanisms linking chronic pain and anxiety, with the aim of identifying molecular targets for TENS-based treatment of their comorbidity.

## Data Availability

The original contributions presented in this study are included in the article/Supplementary Material. Further inquiries can be directed to the corresponding authors.

## Supplementary Materials

The following supporting information can be downloaded at: https://www.mdpi.com/article/10.3390/biomedicines14030670/s1, Figure S1. Schematic diagram of the experimental setup for in vivo calcium imaging. Figure S2. Schematic diagram of in vivo calcium imaging in Mrgprb4-GCaMP6s transgenic mice during TENS. Figure S3. Immunofluorescence analysis of Mrgprb4-lineage neurons in the dorsal root ganglion (DRG) and skin.

## Abbreviations

TRPV1	Transient receptor potential vanilloid 1
EA	Electroacupuncture
CFA	Complete Freund’s Adjuvant
Mrgprb4	Mas-related G-protein-coupled receptor b4
TENS	Transcutaneous electrical nerve stimulation
ST36	Zusanli
DRG	Dorsal root ganglia
AUC	Area under the curve
DA	Dopamine
SPB	Spinoparabrachial
Gpr83	G protein-coupled receptor 83
CON	Control
ABL	Ablation
VTA	Ventral tegmental area
NAc	Nucleus accumbens

## References

[1] Price D.D. (2000). Psychological and neural mechanisms of the affective dimension of pain. Science.

[2] Bouhassira D. (2019). Neuropathic pain: Definition, assessment and epidemiology. Rev. Neurol..

[3] Bandelow B. (2015). Generalized Anxiety Disorder and Pain. Mod. Trends Pharmacopsychiatry.

[4] Velly A.M., Mohit S. (2018). Epidemiology of pain and relation to psychiatric disorders. Prog. Neuropsychopharmacol. Biol. Psychiatry.

[5] Tsang A., Von Korff M., Lee S., Alonso J., Karam E., Angermeyer M.C., Borges G.L., Bromet E.J., Demytteneare K., de Girolamo G. (2008). Common chronic pain conditions in developed and developing countries: Gender and age differences and comorbidity with depression-anxiety disorders. J. Pain.

[6] Jones J., Correll D.J., Lechner S.M., Jazic I., Miao X., Shaw D., Simard C., Osteen J.D., Hare B., Beaton A. (2023). Selective Inhibition of Na(V)1.8 with VX-548 for Acute Pain. N. Engl. J. Med..

[7] Mecum N.E., Russell R., Lee J., Sullivan C., Meng I.D. (2021). Optogenetic Inhibition of Nav1.8 Expressing Corneal Afferents Reduces Persistent Dry Eye Pain. Investig. Ophthalmol. Vis. Sci..

[8] Zhang B.Y., Zhang Y.L., Sun Q., Zhang P.A., Wang X.X., Xu G.Y., Hu J., Zhang H.H. (2020). Alpha-lipoic acid downregulates TRPV1 receptor via NF-κB and attenuates neuropathic pain in rats with diabetes. CNS Neurosci. Ther..

[9] Barton N.J., McQueen D.S., Thomson D., Gauldie S.D., Wilson A.W., Salter D.M., Chessell I.P. (2006). Attenuation of experimental arthritis in TRPV1R knockout mice. Exp. Mol. Pathol..

[10] Cavanaugh D.J., Lee H., Lo L., Shields S.D., Zylka M.J., Basbaum A.I., Anderson D.J. (2009). Distinct subsets of unmyelinated primary sensory fibers mediate behavioral responses to noxious thermal and mechanical stimuli. Proc. Natl. Acad. Sci. USA.

[11] Liao H.Y., Lin Y.W. (2021). Electroacupuncture Attenuates Chronic Inflammatory Pain and Depression Comorbidity through Transient Receptor Potential V1 in the Brain. Am. J. Chin. Med..

[12] Du L., Cheng H., Cui X., Cao Q., Li X., Wang S., Wang X., Liu Y., Zhu B., Gao X. (2025). Mrgprb4-lineage neurons indispensable in pressure induced pleasant sensation are polymodal. iScience.

[13] Liu K., Zhu B. (2023). Significance of pleasant touch and state-of-the-art neuroscience technologies in acupuncture research. Acupunct. Herb. Med..

[14] Elias L.J., Succi I.K., Schaffler M.D., Foster W., Gradwell M.A., Bohic M., Fushiki A., Upadhyay A., Ejoh L.L., Schwark R. (2023). Touch neurons underlying dopaminergic pleasurable touch and sexual receptivity. Cell.

[15] Hylands-White N., Duarte R.V., Raphael J.H. (2017). An overview of treatment approaches for chronic pain management. Rheumatol. Int..

[16] Rocchio R.J., Ward K.E. (2021). Intranasal Ketamine for Acute Pain. Clin. J. Pain.

[17] Dailey D.L., Vance C.G.T., Rakel B.A., Zimmerman M.B., Embree J., Merriwether E.N., Geasland K.M., Chimenti R., Williams J.M., Golchha M. (2020). Transcutaneous Electrical Nerve Stimulation Reduces Movement-Evoked Pain and Fatigue: A Randomized, Controlled Trial. Arthritis Rheumatol..

[18] Dailey D.L., Rakel B.A., Vance C.G.T., Liebano R.E., Amrit A.S., Bush H.M., Lee K.S., Lee J.E., Sluka K.A. (2013). Transcutaneous electrical nerve stimulation reduces pain, fatigue and hyperalgesia while restoring central inhibition in primary fibromyalgia. Pain.

[19] Sabino G.S., Santos C.M., Francischi J.N., de Resende M.A. (2008). Release of endogenous opioids following transcutaneous electric nerve stimulation in an experimental model of acute inflammatory pain. J. Pain.

[20] DeSantana J.M., Santana-Filho V.J., Guerra D.R., Sluka K.A., Gurgel R.Q., da Silva W.M. (2008). Hypoalgesic effect of the transcutaneous electrical nerve stimulation following inguinal herniorrhaphy: A randomized, controlled trial. J. Pain.

[21] Osiri M., Welch V., Brosseau L., Shea B., McGowan J., Tugwell P., Wells G. (2000). Transcutaneous electrical nerve stimulation for knee osteoarthritis. Cochrane Database Syst Rev..

[22] Schuster G.D., Infante M.C. (1980). Pain relief after low back surgery: The efficacy of transcutaneous electrical nerve stimulation. Pain.

[23] Tanaka M., Vécsei L. (2024). From Lab to Life: Exploring Cutting-Edge Models for Neurological and Psychiatric Disorders. Biomedicines.

[24] Cebalo N., Negovetić Vranić D., Bašić Kes V. (2020). The Effect of Transcutaneous Electric Nerve Stimulation (TENS) on Anxiety and Fear in Children Aged 9-14 Years. Acta Stomatol. Croat..

[25] Ferrara J., Stamey W., Strutt A.M., Adam O.R., Jankovic J. (2011). Transcutaneous electrical stimulation (TENS) for psychogenic movement disorders. J. Neuropsychiatry Clin. Neurosci..

[26] Al-Zamil M., Minenko I.A., Kulikova N.G., Mansur N., Nuvakhova M.B., Khripunova O.V., Shurygina I.P., Topolyanskaya S.V., Trefilova V.V., Petrova M.M. (2023). Efficiency of Direct Transcutaneous Electroneurostimulation of the Median Nerve in the Regression of Residual Neurological Symptoms after Carpal Tunnel Decompression Surgery. Biomedicines.

[27] Chen L., Tang J., White P.F., Sloninsky A., Wender R.H., Naruse R., Kariger R. (1998). The effect of location of transcutaneous electrical nerve stimulation on postoperative opioid analgesic requirement: Acupoint versus nonacupoint stimulation. Anesth. Analg..

[28] Chen Z., Zhang C., Song X., Cui X., Liu J., Ford N.C., He S., Zhu G., Dong X., Hanani M. (2022). BzATP Activates Satellite Glial Cells and Increases the Excitability of Dorsal Root Ganglia Neurons in Vivo. Cells.

[29] Liu B., Qiao L., Liu K., Liu J., Piccinni-Ash T.J., Chen Z.F. (2022). Molecular and neural basis of pleasant touch sensation. Science.

[30] Zhongren L. (2007). Experimental Acupuncturology.

[31] Gao X., Han S., Huang Q., He S.Q., Ford N.C., Zheng Q., Chen Z., Yu S., Dong X., Guan Y. (2021). Calcium imaging in population of dorsal root ganglion neurons unravels novel mechanisms of visceral pain sensitization and referred somatic hypersensitivity. Pain.

[32] Chen Z., Huang Q., Song X., Ford N.C., Zhang C., Xu Q., Lay M., He S.Q., Dong X., Hanani M. (2022). Purinergic signaling between neurons and satellite glial cells of mouse dorsal root ganglia modulates neuronal excitability in vivo. Pain.

[33] Liu K., Liu Y., Li X., Wang S., Wang X., Zhang Z., Gao X. (2024). In Vivo Calcium Imaging of Dorsal Root Ganglia Neurons’ Response to Somatic and Visceral Stimuli. J. Vis. Exp..

[34] Lampe G.N. (1978). Introduction to the use of transcutaneous electrical nerve stimulation devices. Phys. Ther..

[35] Smith T.J., Wang E.J., Loprinzi C.L. (2023). Cutaneous Electroanalgesia for Relief of Chronic and Neuropathic Pain. N. Engl. J. Med..

[36] Masson E.A., Veves A., Fernando D., Boulton A.J. (1989). Current perception thresholds: A new, quick, and reproducible method for the assessment of peripheral neuropathy in diabetes mellitus. Diabetologia.

[37] Reeve A.J., Walker K., Urban L., Fox A. (2000). Excitatory effects of galanin in the spinal cord of intact, anaesthetized rats. Neurosci. Lett..

[38] Dufour A., Guergova S., Pebayle T., Touzalin-Chretien P. (2011). On the selective activation of unmyelinated C-fibers using sinusoidal electrical stimulation: An ERP study. Clin. Neurophysiol..

[39] Wright A. (2012). Exploring the evidence for using TENS to relieve pain. Nurs. Times.

[40] Liu S., Long S.S., Li F., Yang H., Pu S., Du D., Luo X., Zhang Y.Q., Han Q. (2025). Neural basis of transcutaneous electrical nerve stimulation for neuropathic pain relief. Neuron.

[41] Lee J.H., Choi Y.H., Choi B.T. (2005). The anti-inflammatory effects of 2 Hz electroacupuncture with different intensities on acute carrageenan-induced inflammation in the rat paw. Int. J. Mol. Med..

[42] Guo Z., Wei N., Ye R., Sun T., Qiu S., Shao X., Ge X., Guan L., Fang J., Fang J. (2024). Map activation of various brain regions using different frequencies of electroacupuncture ST36, utilizing the Fos-CreER strategy. Acupunct. Herb. Med..

[43] Li Y.J., Du W.J., Liu R., Zan G.Y., Ye B.L., Li Q., Sheng Z.H., Yuan Y.W., Song Y.J., Liu J.G. (2023). Paraventricular nucleus-central amygdala oxytocinergic projection modulates pain-related anxiety-like behaviors in mice. CNS Neurosci. Ther..

[44] Guo H., Hu W.C., Xian H., Shi Y.X., Liu Y.Y., Ma S.B., Pan K.Q., Wu S.X., Xu L.Y., Luo C. (2024). CCL2 Potentiates Inflammation Pain and Related Anxiety-Like Behavior Through NMDA Signaling in Anterior Cingulate Cortex. Mol. Neurobiol..

[45] McCarson K.E., Fehrenbacher J.C. (2021). Models of Inflammation: Carrageenan- or Complete Freund’s Adjuvant (CFA)-Induced Edema and Hypersensitivity in the Rat. Curr. Protoc..

[46] Chen J., Song Y., Yang J., Zhang Y., Zhao P., Zhu X.J., Su H.C. (2013). The contribution of TNF-α in the amygdala to anxiety in mice with persistent inflammatory pain. Neurosci. Lett..

[47] Melzack R., Wall P.D. (1965). Pain mechanisms: A new theory. Science.

[48] Lu Y., Perl E.R. (2003). A specific inhibitory pathway between substantia gelatinosa neurons receiving direct C-fiber input. J. Neurosci..

[49] Zarrindast M.R., Khakpai F. (2015). The Modulatory Role of Dopamine in Anxiety-like Behavior. Arch. Iran. Med..

[50] Choi S., Hachisuka J., Brett M.A., Magee A.R., Omori Y., Iqbal N.U., Zhang D., DeLisle M.M., Wolfson R.L., Bai L. (2020). Parallel ascending spinal pathways for affective touch and pain. Nature.

[51] Tzschentke T.M. (2007). Measuring reward with the conditioned place preference (CPP) paradigm: Update of the last decade. Addict. Biol..

[52] Vrontou S., Wong A.M., Rau K.K., Koerber H.R., Anderson D.J. (2013). Genetic identification of C fibres that detect massage-like stroking of hairy skin in vivo. Nature.

[53] Yang H., de Jong J.W., Cerniauskas I., Peck J.R., Lim B.K., Gong H., Fields H.L., Lammel S. (2021). Pain modulates dopamine neurons via a spinal-parabrachial-mesencephalic circuit. Nat. Neurosci..

[54] Coizet V., Dommett E.J., Klop E.M., Redgrave P., Overton P.G. (2010). The parabrachial nucleus is a critical link in the transmission of short latency nociceptive information to midbrain dopaminergic neurons. Neuroscience.

[55] Majumdar S., Prajapati S.K., Dande A., Yata V.K., Choudhary K., Peraman R., Kumar N., Krishnamurthy S. (2026). Integrative Proteomics Reveal Neuroimmune and Dopaminergic Alterations Across the Nociceptive Neuraxis in Neuropathic Pain. Cells.

[56] DeGroot S.R., Zhao-Shea R., Chung L., Klenowski P.M., Sun F., Molas S., Gardner P.D., Li Y., Tapper A.R. (2020). Midbrain Dopamine Controls Anxiety-like Behavior by Engaging Unique Interpeduncular Nucleus Microcircuitry. Biol. Psychiatry.

[57] Calipari E.S. (2020). Dopamine Release in the Midbrain Promotes Anxiety. Biol. Psychiatry.

