# Supplementary material for: Mrgprb4-Lineage Neurons Participate in the Intervention of TENS Effects on Chronic Pain and Anxiety-like Symptoms in an Inflammatory Pain Mouse Model
Source: Biomedicines. 2026 Mar 15;14(3):670. doi: 10.3390/biomedicines14030670 (PMC13024121; doi:10.3390/biomedicines14030670)
Supplement: Supplementary file 1 [file biomedicines-14-00670-s001.zip › biomedicines-4120197-supplementary.pdf]

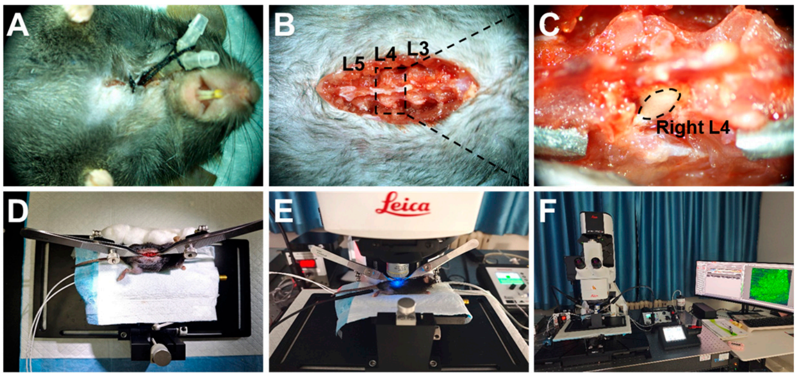

Fig. S1 Schematic diagram of the experimental setup for in vivo calcium imaging. **A** Tracheal intubation. **B-C** Exposure of the L4 dorsal root ganglion (DRG). **D** Immobilization of the L4 DRG. E-F In vivo calcium imaging of the DRG under the microscope.

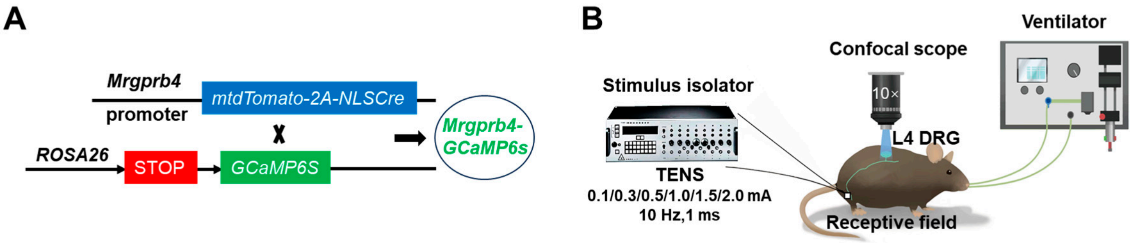

Fig. S2 Schematic diagram of in vivo calcium imaging in Mrgprb4-GCaMP6s transgenic mice during TENS. **A** Breeding strategy for generating Mrgprb4-GCaMP6s transgenic mice. **B** Setup for *in vivo* calcium imaging in the L4 DRG during TENS application at varying intensities to the receptive field.

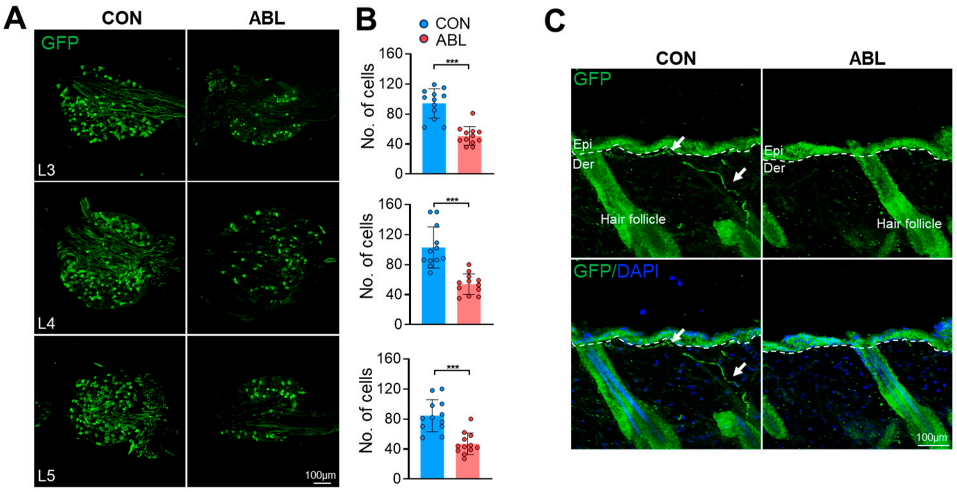

Fig. S3 Immunofluorescence analysis of Mrgprb4-lineage neurons in the dorsal root ganglion (DRG) and skin. **A** Representative immunofluorescence images of L3-L5 DRG sections from a control (left) and

a Mrgprb4-neuron-ablated (right) Mrgprb4Cre; Rosa26-ChR2-EYFP mouse. Scale bar = 100 μm. **B** Quantification of Mrgprb4-positive neurons in the DRG from control and ablated mice (n = X). **C** Representative immunofluorescence images of skin sections from a control (left) and a Mrgprb4-neuron-ablated (right) Mrgprb4Cre; Rosa26-ChR2-EYFP mouse. Scale bar = 100 μm.
